# Clinical Manifestation of Juvenile and Pediatric HD Patients: A Retrospective Case Series

**DOI:** 10.3390/brainsci10060340

**Published:** 2020-06-03

**Authors:** Jannis Achenbach, Charlotte Thiels, Thomas Lücke, Carsten Saft

**Affiliations:** 1 Department of Neurology, Huntington Centre North Rhine-Westphalia, St. Josef-Hospital Bochum, Ruhr-University Bochum, 44791 Bochum, Germany; carsten.saft@rub.de; 2 Department of Neuropaediatrics and Social Paediatrics, University Children’s Hospital, Ruhr-University Bochum, 44791 Bochum, Germany; charlotte.thiels@rub.de (C.T.); luecke.thomas@ruhr-uni-bochum.de (T.L.)

**Keywords:** juvenile Huntington’s disease, pediatric Huntington’s disease, early-onset Huntington’s disease, case series

## Abstract

Background: Studies on the clinical manifestation and course of disease in children suffering from Huntington’s disease (HD) are rare. Case reports of juvenile HD (onset ≤ 20 years) describe heterogeneous motoric and non-motoric symptoms, often accompanied with a delay in diagnosis. We aimed to describe this rare group of patients, especially with regard to socio-medical aspects and individual or common treatment strategies. In addition, we differentiated between juvenile and the recently defined pediatric HD population (onset < 18 years). Methods: Out of 2593 individual HD patients treated within the last 25 years in the Huntington Centre, North Rhine-Westphalia (NRW), 32 subjects were analyzed with an early onset younger than 21 years (1.23%, juvenile) and 18 of them younger than 18 years of age (0.69%, pediatric). Results: Beside a high degree of school problems, irritability or aggressive behavior (62.5% of pediatric and 31.2% of juvenile cases), serious problems concerning the social and family background were reported in 25% of the pediatric cohort. This includes an attempted rape and robbery at the age of 12, as problems caused by the affected children, but also alcohol-dependency in a two-year-old induced by a non-HD affected stepfather. A high degree of suicidal attempts and ideations (31.2% in pediatric and 33.3% in juvenile group) was reported, including drinking of solvents, swallowing razor blades or jumping from the fifth floor with following incomplete paraparesis. Beside dopaminergic drugs for treatment of bradykinesia, benzodiazepines and tetrabenazine for treatment of dystonia, cannabinoids, botulinum toxin injection and deep brain stimulation were used for the improvement of movement disorders, clozapine for the treatment of tremor, and dopa-induced hallucinations and zuclopenthixole for the treatment of severe aggressive behavior. Conclusions: Beside abnormalities in behavior from an early age due to HD pathology, children seem to have higher socio-medical problems related to additional burden caused by early affected parents, instable family backgrounds including drug abuse of a parent or multiple changes of partners. Treatment required individualized strategies in many cases.

## 1. Introduction 

Huntington’s disease (HD) is an autosomal-dominant hereditary neurodegenerative progressive disorder usually with a most common onset at the age of 30–50 years [1,2]. Nevertheless, motor onset can occur at every age and onsets ≤ 20 years of age are traditionally classified as juvenile HD (JHD) [3]. Recent research suggested the redefining of the term of younger HD patients and to use the term “pediatric” instead of juvenile Huntington’s disease for those younger than 18 years, since the definition of juvenile HD (JHD) was found to be blurred and used in different ways [4]. The onset is thereby defined as the presence of unequivocal clinical motor signs (>99% confidence with a diagnostic confidence level (DCL) of four on the “Unified Huntington’s Disease Rating Scale” (UHDRS) [5]) caused by HD. Especially in younger patients, motor symptoms may present typically with bradykinesia, dystonia, but also with myoclonus or tremor. The cohort of JHD was described with varieties of motor- and non-motor specific characteristics, thus demands were made for adapting common rating scales, usually to access disease-manifestation in adult HD, for use in children [6].

Case series reports on psychiatric and cognitive nonspecific deficits often accompanied a misdiagnosis or delays in HD diagnosis. JHD patients having an earlier onset also are described to have a longer delay between symptoms and diagnosis of HD [7]. Although the detectable genetic cause with an expansion of cytosine- adenine- guanine (CAG)-trinucleotide repeats in the huntingtin gene (HTT) on chromosome four is obvious to access [1,8], repeat expansions higher than 60–70 CAGs are described as causing a juvenile and more bradykinetic HD phenotype and were frequently detected in juvenile cases described in earlier research [7,8,9,10]. Although the genetic cause is unequivocal, characteristics of the clinical disease manifestation, especially in children, are manifold. HD is described as a complex disease with heterogeneous challenges and progressive loss of dependency and increasing disability. For the care of HD patients, several researchers recommend multidisciplinary approaches which are required and include the family, social workers, therapists and physicians to maintain quality of life and to decrease psychosocial problems [11]. For pediatric and juvenile HD, the support not only for children but also for parents or even the whole social system including schools, might be helpful. Psychosocial implications in these settings are described as wide ranged. Former research describes the role of the family and caregivers as burdensome due to disappointing experiences with social and health services, the dissatisfaction of being a caregiver, concern about children, and the loss of needed help from the social or family background due to increasing care for the HD patient and social withdrawal [12]. The unaffected family caregiver is thereby described to need the most support, attention, and help [12]. Many important aspects concerning these socio-medical and psychosocial backgrounds in HD are described [13]. Challenging ethical, social, and legal issues for HD patients, health care professionals, and caregivers are caused by the progressive disease [14]. Most published data consider HD patients and their influence on the social environment. However, further research is necessary regarding social aspects of support and the effects of the caregiver’s behavior on the disease and the behavior of the patient and how these interactions affect relations in HD families [15].

The prevalence of HD varies between different geographical regions [16]. Systematic reviews indicated global variations, with an overall prevalence of 5.7 per 10,0000 persons, describing a lower incidence in Asia compared to Europe and North America [3,17]. However, more recently a substantial higher prevalence of HD in the UK (12.3 per 10,0000) and also in Germany was reported, with 9.3 per 10,0000 inhabitants using data of four million insured persons, probably caused by more accurate diagnoses and an improved life expectancy [18,19].The small cohort of juvenile HD patients is described with an equivocal estimated prevalence ranging between 1–9.6% of all HD cases in studies and meta-analyses estimating 4.92% to 6% of all HD patients being juvenile [4,20,21,22,23].

Although many important aspects about juvenile HD have been described, no case reports about the recently defined pediatric cohort or comparing research of pediatric and juvenile HD in the boundary of typical characteristics in adult HD were described [4]. Describing the different phenotypes of the clinical manifestation in HD and especially early-onset HD is not only important for the investigation of potential underlying and diverse mechanisms of pathophysiology but primarily for the adapting of different symptomatic therapeutic options. Hereby an exact assessment and rating of predominant symptoms can be crucial because the symptomatic therapy needs to be adapted to the type and extent of individual findings and adjusted frequently during the individual course of the disease [24]. Contrary to treatment of chorea, in a more bradykinetic phenotype, as described for JHD, dopamine agonists may be effective [24]. Case reports and small studies report on an improvement after the dopamine agonist pramipexole or after dosing of medication with L-dopa and amantadine [24,25]. Beside these options for symptomatic therapies, there are no disease-modifying/slowing options or therapeutic options with neuroprotective effects available at the moment [26].

The aim of this case report series is to describe the rare group of early-onset patients, especially in regard to socio-medical aspects and individual or common treatment strategies. In addition, we differentiated between juvenile and the recently defined pediatric HD population (onset < 18 years).

## 2. Patients and Methods

To classify a cohort of the comparatively very rare pediatric and juvenile HD-patients in more detail, we investigated a retrospective analysis of data from our Huntington Centre North Rhine- Westphalia (NRW). Since its establishment in the year 1995, we have had 25 years of clinical and research experience concerning the treatment and care of adult and early-onset patients suffering from HD. A University Children’s Hospital for Neuropaediatrics and Social Paediatrics is affiliated as a part of our institution. The affiliation of the department of Neuropaediatrics and Social Paediatrics to the Huntington Centre NRW for the diagnosis and treatment of HD children is well known among German pediatricians as a result of talks held at pediatric congresses and publications in pediatric journals. Moreover, this cooperation is well known among the German patient support organization, further admissions take place after molecular diagnostic testing in the department of human genetics as part of the Huntington Centre NRW. A rooming in together with a parent was possible when treating children as inpatients.

We analyzed data from our internal digital quality management and hospital information system as well as archived medical letters and examination reports. Apart from this, additional data were collected from archived admission books of the outpatient and inpatient clinic and analyzed, (i) to receive information about a prevalence of juvenile and pediatric HD patients in our clinic, and (ii) to especially evaluate clinical patient-related information for presenting case reports and fundamental correlations of the heterogeneous clinical pictures in early-onset HD.

A special focus was set to describe challenging situations concerning the diagnosis and pharmaceutical treatment of affected patients compared to adult patients as well as challenging situations coming from the socio-medical environment of individual patients. Data concerning socio-medical information were anonymized, analyzed, and based on socio-medical anamnesis in the medical reports. No information was available coming from other sources, such as criminal reports or court proceedings. The investigation was confirmed by our local ethics committee (registration number 20-6892-BR) who agreed on the retrospective anonymized data analysis and publication of information coming from our clinical data management system and medical letters.

## 3. Results

Considering the last 25 years of the Huntington Centre NRW, we identified 2593 individual patients suffering from HD and presenting in our outpatient and inpatient clinic for seeking medical advice or treatment.

Out of these 2593 patients, 32 individuals (Table 1) in total were identified with an early-onset of a manifest disease when younger than 21, which corresponds to a ratio of 1.23%. Children from all over Germany were admitted to the hospital. Dividing the 32 early-onset patients into pediatric and juvenile HD revealed 18 patients who were classified as pediatric (onset < 18 years of age) and 14 more being juvenile (18 < onset < 21 years of age). Therewith, the proportion of pediatric out of all HD patients presenting in our centre was identified as 0.69%.

Considering the described cohort of early-onset patients, 59.4% appeared to be male patients. In the pediatric cohort, the rate of male patients (72.2%), corresponding to 13 out of 18 patients, was higher than in the juvenile cohort, where a lower rate of 42.9% (six out of 14 patients) appeared to be male.

Analyzing the early-onset cohort more closely revealed, 16 cases with a paternal inheritance model, seven cases with a maternal inheritance, two cases without any HD in the anamnesis of the accompanied parents or rest of the family anamnesis, and seven cases without given data from family anamnesis. As a reason for missing family history, detailed information from medical reports were not available any more in seven cases because reports were destroyed after the minimum of 10 years duty of archiving. The 23 cases with reported data from the family anamnesis therewith revealed a paternal inheritance of 69.6% in the monitored cohort.

The reported CAG repeat length of all early-onset patients (*n* = 25) revealed a median expanded allele length of 63. After diving into the different groups of pediatric (*n* = 14; M = 67.3; SD = 11.6) compared to juvenile HD patients (*n* = 11; M = 57.4; SD = 5.6), independent *t*-test (IBM^®^ SPSS^®^ Statistic V25, IBM Corp., Armonk, NY, USA) demonstrated significantly higher CAG repeats in the pediatric group (*t*(19.6) = −2,8, *p* = 0.011).

In the juvenile cohort, a mean age of onset (*n* = 14) was identified as 19.3 years (SD 0.9) and the age of diagnosis (*n* = 9) on average identified as 23.2 years (SD 1.2) (Table 2). The pediatric cohort revealed a mean age of onset (*n* = 16) of 10.3 years (SD 4.1) and an average age of diagnosis (*n* = 17) of 13.5 years (SD 3.8). Describing the cumulated percentages in the pediatric group revealed that 50% of the cohort had an onset ≤ 9 years of age and 75% of the cohort ≤ 13 years of age. Of the pediatric group, 52.9% was diagnosed with an age ≤ 13 years and 76.5% with an age ≤ 16 years.

Comparing these median reported ages in juvenile and pediatric groups using independent t-tests demonstrated a significantly earlier onset (*t*(16.6) = 8.4, *p* ≤ 0.001) and diagnosis (*t*(21.4) = 9.1, *p* ≤ 0.001) in the pediatric group.

The mean times measured in years between age of onset and diagnosis revealed no significant difference between the pediatric (*n* = 15; M = 3.5; SD 1.9) and the juvenile group (*n* = 9; M = 3.0; SD 1.0) in the independent *t*-test (*t*(22) = −0.8, *p* = 0.411).

The motoric components of the disease manifestation revealed especially hypokinetic-bradykinetic compounds and rigidity being present in the majority of the pediatric cohort (reported in 11 out of 16 cases: 1, 2, 3, 5, 6, 7, 8, 9, 13, 14, 20) and also present in five out of nine cases with juvenile HD (cases 15, 18, 20, 23,24).

Six out of 16 pediatric cases (case 1, 2, 7, 8, 9, 20) revealed the presence of dystonia and therewith in 37.5% of the pediatric collective compared to 22.2% of juvenile cases (two of nine cases; cases 16, 24).

Hyperkinetic movement disorders in terms of chorea were present in cases 16, 18, 19, 21, 24 (all juvenile) and slightly in cases 22, 24 (both pediatric), which represents 55.6% of the juvenile and 12.5% of the described pediatric cohort. Another hyperkinetic movement disorder, myoclonus, was identified in two out of nine juvenile phenotypes (22.2% of the juvenile cohort).

Out of 16 pediatric patients, we identified six patients who were diagnosed with epilepsy, which corresponds to 37.5% of that pediatric cohort. In comparison, we identified two out of nine patients with epilepsy in the juvenile cohort (22.2% of the cohort).

Six out of 16 pediatric patients presented with tremor in their clinical assessments, which corresponds to 37.5% of the cohort. In the juvenile cohort one patient suffered from tremor (11.1% of the cohort).

Changing and difficulties of speech were present in cases 4, 6, 7, 13 (all pediatric) and case 17 (juvenile), and therewith relevant in 25% of the pediatric and 11.1% of the juvenile cohort.

Concerning the diversity of psychiatric problems, aggression and irritability was reported in 10 out of 16 pediatric cases (cases 2, 4, 5, 6, 11, 12, 13, 14, 22, 25), which corresponds to a ratio of 62.5%, and in three juvenile cases (cases 17, 18, 19), which is a ratio of 33.3% in the juvenile cohort. Suicidal ideations or attempts were described in eight cases in total, whereby five cases were pediatric (cases 2, 3, 5, 18, 22) and three cases were juvenile (cases 15, 17, 19), which reports a ratio of 31.2% in the pediatric and 33.3% in the juvenile group.

The presence of apathy in cases 2, 25 (both pediatric) as well as obsessive behavior in case 19 (juvenile) and 25 (pediatric) were described as other psychiatric symptoms.

The main aspects of social pediatric characteristics especially included problems in school, described in five pediatric cases (cases 2, 3, 4, 5, 6) and one juvenile case (case 7) as frequent initial symptoms, especially in the pediatric group and also accompanied with a cognitive decline (cases 1, 2, 9, 17).

Furthermore, other patients showed criminal behavior characteristics (case 6) as well as substantial problems with alcohol and drug abuse (cases 11, 13, 15) and serious problems concerning the social background and family, with violation and conflicts in the family (all pediatric cases 3, 11, 12, 14), which equates a ratio of 25% in the pediatric cohort.

Considering diagnostic interventions, five out of 16 pediatric patients (31.2%) and one juvenile case (21) had an MRI, whereby only in one case was an inconspicuous result reported.

## 4. Discussion

Juvenile HD is described as a very rare manifestation of HD with an estimated prevalence of 1–9.6% of all HD cases in studies and meta-analyses estimate 4.92% to 6% of all HD patients being juvenile [4,12,13,14,15]. Out of 2593 individual HD patients treated within the last 25 years in the Huntington Centre NRW, 32 subjects were analyzed with an early onset younger than 21 years. This relates to a proportion of 1.23%. This is within the range of published data. Several problems arising from the terminology JHD are currently being discussed. Many patients seem to be called juvenile because of their more bradykinetic phenotype even if they are older than 21 [21,27,28]. Thus, in JHD the bradykinetic phenotype can be seen as being most characteristic, which is usually associated with a more dopaminergic therapy. However, there seems to be a large cohort of HD patients who are already adults suffering from a more predominant bradykinetic-rigid clinical phenotype, called JHD. Some of them perhaps even had a motor onset beyond 20 years of age and are called JHD only because of their more bradykinetic symptoms [29]. This might explain the high range for estimation of prevalence of 1–9.6% and also explain why the prevalence in our cohort does not correspond with the higher mean described above and classified in the lower range since we used the strict definition of an age below 21 year for our collective. This difference is even more obvious if the more recently definition of pediatric HD is used, with only 0.69% of all cases. This remarkably low rate might be caused due to fewer admissions of early-onset HD cases to our centre. However, as part of our institution, a university children’s hospital for Neuropaediatrics and Social Paediatrics is affiliated and well known by the German pediatricians society and patient support organisation. Since to the best of our knowledge, this is the only known cooperation like this in Germany and the identified cases were submitted nationwide, we would expect a bias to even more JHD cases and not less in our cohort.

As a first aspect to discuss in our case report series we detected several findings concerning socio-medical aspects not only caused by HD pathology but also caused by early affected parents, instable family backgrounds, including drug abuse by a parent or multiple changes of partners. The results of our research are in line with earlier findings. Massive burdens for caregivers, HD families and especially for the patient arise from socio-medical problems in an observed mutual interplay of the social surrounding and the affected person [12].

That implements not only the pharmacological treatment of patients but also more socio-medical supports like the organization of a school accompaniment, support by a social worker and support of the non- affected parent including psychotherapy in order to reach and maintain a certain degree of education. Especially, problems in school were often described as initial symptoms in many cases, they were also accompanied by cognitive decline. In this context, the establishing of the diagnosis HD was extremely helpful for most cases in our experience since it was possible to reduce learning pressure in school and define new targets for education in discussion with the school. Children in most cases were not depressed or burdened by the establishing of the HD diagnosis, but relieved by finding an explanation for their difficulties at school and by defining new, more social aspects of participating in school visits. This is even more important since a delay in diagnosis is described in our collective and also published data in many cases [30] as one reason for late diagnosis the resistance of parents was reported. Following our experience, we would rather recommend an earlier diagnosis than a delay in diagnosis to parents in order to reduce the burdens of their children in social-medical aspects. As another reason for late diagnosis, a lack of knowledge among health care professionals is discussed, possibly also caused by unsuspectingness about the heterogeneous motoric symptomatic with a more bradykinetic motor phenotype in early- onset HD. In many of our cases, expensive and stressful or even painful diagnostic interventions like lumbar punctures were performed even if family history of HD was positive. In addition, some cases were initially misdiagnosed and mistreated as attention deficit hyperactivity disorder or borderline disease. Thus, we can support former described findings of delayed diagnosis in this subgroup [30]. From our perspective it is decisive to enable HD patients and families a continuous care through a specialized centre. For this purpose, participating in a prospective registry study (e.g., ENROLL-HD) might enable annual visits, review of socio-medical aspects, and the family anamnesis. If an affected HD family member or caregiver is in contact with a specialized centre and reports difficulties with a child, an investigation of this child should be offered. Additionally, index patients should be asked specifically about their own children and whether there are any abnormalities observed. A multidisciplinary setting including an early involvement of neurologists and neuropaediatricians is crucial and extremely helpful in our view. If there are typical HD symptoms, in most cases being investigated in a centre, diagnosis is not substantially delayed in our experience. However, if children are not initially investigated in a centre, we observed long delays and multiple unnecessary and stressful examinations including lumbar puncture and others in many cases. This might be due to several reasons: the lack of knowledge about more bradykinetic symptoms, concerns regarding performing genetic testing in a child with a lack of knowledge about the differential diagnostic-testing procedure, or concerns to make such a severe diagnosis as HD. Thus, the only way to avoid delays in diagnosis in these cases might be through the participation of professionals from a centre in meetings of patient-organisations, participation in congresses for professionals (e.g., neurologists or neuropaediatricians), public relations work about HD, and the offer of training courses for the specialized public. More important, from our experience, when making the diagnosis of HD in children is that almost all children were more relieved by the diagnosis than burdened. Regardless of the therapy with drugs, most children had a relief if their existing problems in school, in sports or with friends could be explained due to the HD diagnose. For example, subsequent changes or help in school setting reduced stress and declined fear of failure. Our impression is, that children, as in other severe diagnoses like cancer, seem to cope with the diagnosis well in most cases whereas diagnosis is often more difficult for the parents.

As a second aspect, it is important to distinguish between the heterogeneous movement disorders in early- onset HD, with bradykinesia, dystonia, tremor or myoclonic hyperkinesia in order to achieve a beneficial pharmacological treatment [31,32,33]. Many of our cases were treated with dopaminergic drugs or amantadine for an improvement of bradykinesia, benzodiazepines or tetrabenazine was used for treatment of dystonia with beneficial effects. In single severe cases, cannabinoids and deep brain stimulation (DBS) was used for more generalized dystonia or botulinum toxin- injection for focal dystonia. For DBS only, intermittent positive effects and only mild beneficial effects after treatment with trihexyphenidyl were observed [33]. A relatively high amount of 37.5% of the pediatric cohort and 11.1% of the juvenile cohort, respectively, suffered from tremor. In most cases tremor was treated with dopa-agonists like pramipexole, in one case an excellent benefit was observed after treatment with clozapine with an initial indication of dopa-induced hallucination [34]. Valproic acid was used for treatment of epilepsy, as a mood stabilizer but also for treatment of myoclonic hyperkinesia in two cases [31]. As listed in our reports, in many cases a combination of medication or changes in medication in the course of the disease was necessary.

The occurrence of epilepsy with seizures in JHD is described as an additional important clinical feature [7,10,35]. Although not much is known about why epilepsy occurs more in JHD than in adult- onset HD, retrospective studies report on a very frequent occurrence of 38% in a collective of juvenile HD patients, which corresponds to a rate of 37.5% in our collective [36]. Valproic acid was used for treatment in many cases but also levetiracetam, lamotrigine and benzodiazepines. No case of increased irritability as a known side-effect was observed regarding treatment with levetiracetam.

As another clinical feature, a delay of speech development was observed in five cases and therewith present in 25% of the pediatric cohort. Remarkably, the speech development delay and a consequent logopaedic therapeutic trial was even described in advance of other clinical symptoms or diagnosis of HD. As family anamnesis revealed a younger and older brother without any suspected speech development delay, we assumed a potential organic cause due to HD.

Finally, we observed a very high amount of different psychiatric disturbances like aggression and irritability in 62.5% of the pediatric and 33.3% of the juvenile cohort, respectively. Substantial aggressive and criminal behavior causing serious problems for the family and social network as well as for the patient was observed in case 6 in particular. His mother was suffering from HD and already severely affected at the time of first admission. Besides the potential organic burdens due to the disease, we observed challenging situations for the patient constituted by a socially disadvantaged family situation possibly leading to delinquent behavior due to missing corrective behavior of the father. He was burdened also as the caregiver for the affected mother during that time. Disease-related missing impulse control, an additional impact of the social surrounding, or even further developments of the common puberty-age with a personal-development process can be discussed as being decisive for his behavior. Remarkably, older and younger siblings of this case did not show any delinquent behavior, that is why we assume the described multifactorial reasons including HD related brain changes and not solely the influence of his social background on the described behavior. Suicidal ideations or attempts were described in 31.2% of the pediatric and 33.3% of the juvenile cohort, as well as other psychiatric symptoms, with apathy in two pediatric cases and obsessive behavior in one juvenile and pediatric case [37]. Treatment was effective for most of these cases following guidelines and common psychiatric treatment strategies [27]. Serotonin-selective-reuptake inhibitors (SSRI) and mirtazapine, especially if sleep problems occurred, were the most common medication for treatment of depression. Quetiapine and risperidone were used for the treatment of irritability and aggressive behavior, whereas in single cases zuclopenthixole for the treatment of severe aggressive behavior was also effective [38]. As potential side- effects, bradykinesia and rigidity worsened especially after treatment with risperidone. As a relevant side-effect of therapies with L-dopa or dopa- agonists, hallucinations, especially optical hallucinations, might occur. In five of our 25 cases with complete records, optical, and in one case acoustic, hallucinations were caused due to the dopaminergic treatment and improved after reduction of dosage or with additional neuroleptic treatment [34].

As a limitation, our case reports are based on a retrospective analysis of medical reports and not on standardized implemented scales for accessing of symptoms. Moreover, in seven cases, no additional detailed medical reports were available anymore. However, this more descriptive research approach enabled the depiction of heterogeneous and manifold aspects in early-onset HD which might not be captured in standardized scales, such as the described socio-medical aspects. More in general this is a very rare subgroup of HD, which might limit power for further research in many aspects.

## 5. Conclusions

In summary, beside from early abnormalities in behavior due to HD pathology, children seem to have higher socio-medical problems related to additional burden caused by early affected parents and instable family backgrounds.

## Figures and Tables

**Table 1:** Clinical characteristics and relevant findings of juvenile and/ or pediatric Huntington’s disease (HD) cases from the Huntington Centre, North Rhine-Westphalia (NRW).

First Symptoms	Comorbidities	Predominant Challenging Situation with Special View Regarding Medication
1m→P→80→1.5→9→P		
Change in muscle tone, frequent falls and increasing gait disorder	Epilepsy	Clinical examination (CE): bradykinetic motor-phenotype with increased muscle-tone, rigidity and spastic component, hypokinesia, cognitive decline and dystrophy. Further diagnostics (FD): comprehensive genetic diagnostics with chromosome analysis, analysis of array-based comparative genomic hybridization, analysis of methyl-CPG-binding-protein 2 gene. MRI: Narrowing of the caudate nucleus on both sides and subsequent gene testing with HD diagnosis. Treatment attempts (TA): initially L-dopa/carbidopa attempt because of a suspected tyrosine hydroxylase deficiency. Low-dosed pramipexole retard (bradykinesia) in combination with quetiapine (due to dopa-induced hallucination) lead to an improvement of movement disorder. Clobazam (anxiety- reducing and sleep- inducing) reduced ongoing sleep-disorder and had a positive effect on dystonia and the increased muscle tonus. Levetiracetam used for the treatment of epilepsy. Course of disease (CD): use of dronabinol for palliative medical care with positive effects on muscle tonus, dystonia and body mass index (BMI).
2f→P→73→9→13→P		
Problems in school, pronounced tremor with medium amplitude, gross and fine motoric function		Anamnesis (A): early genetic testing because of positive family history, but retrospective symptoms already exist for the past 4 years. CE: initially hypomimia, lack of arm swinging, cognitive deficits. Socio-medical aspects (SM): re-educated after diagnosis in a special school, which helped enormously. After depression with several suicidal attempts, irritable behavior, lack of impulsion, apathy, schooling no longer possible. Long-term psychiatric ward. FD: electroencephalography (EEG): isolated bi-frontal sharp-wave-like activity and typical epilepsy potentials but no seizures observed while treatment with valproate (for stabilizing irritability). TA: pramipexole (treatment of bradykinesia), venlafaxine (antidepressant), valproate retard, lithium (antidepressant and because of suicidal intent), quetiapine (antipsychotic). In adulthood, worsening of dystonia and spasticity (tiazidine initiated), swallowing and because of improved mood and absence of suicidal tendencies lithium stopped.
3m→P→48→13→14→P		
Problems in school, depression, tremor		A/CE: stressful family situation, father died due to suicide, sister died due to HD. Unspecific symptoms reported (depression, due to HD or family situation?). No genetic testing during the first visit. An additional tremor three months later indicated the genetic testing. FD: diagnostic of cranial MRI, awake, sleep EEG, electrocardiography (ECG) inconspicuous. SM: arrangement with school, compensations for disadvantages helped remaining in class. TA: psychotherapy, occupational therapy but no additional pharmacological therapy necessary at the present. CD: mild bradykinesia in finger tapping without negatively influencing everyday life.
4m→P→81→Infant age→9→P		
Progressive motoric dysfunction, speech problems and problems in school	Epilepsy Severe expressive speech disorder	A: postnatal difficulties in thriving and swallowing. CE: bradykinesia in addition to hyperkinetic restlessness at night. FD: cranial MRI (second year of life) revealed a reduction of brain volume (retrospective evaluated as physiological expansion of the external cerebrospinal fluid spaces in infancy). TA: pramipexole retard (improved bradykinesia), valproate retard (positive influence on irritability), quetiapine (reduction of irritability and hallucinations), L-dopa/carbidopa (positive influence on rigidity) CD: severe swallowing disorder, but percutaneous endoscopic gastrostomy (PEG) rejected.
5f→P→47→10→16→M		
Attention deficit hyperactivity disorder (ADHD), problems in school, depression	Borderline personality disorder	A: initially presented to human genetic specialists by foster parents because of positive HD family history. CE: fine motor skills problems in initial testing. FD: EEG with abnormal findings, without therapeutic consequences. TA: refrain of therapy with dopamine agonists (e.g., pramipexole) due to optical hallucinations. Psychiatric symptoms more severe and main symptoms, motoric symptoms with slight tremor and rigidity. Low-dose risperidone (reduction of irritability and aggressive behavior), quetiapine, venlafaxine (as an antidepressant with only moderate success) and clozapine (because risperidone was not tolerated in higher doses). Clozapine improved emotional instability and psychosis but also tremor. CD: earlier multiple suicide attempts (taking tablets, drinking solvents and swallowing razor blades). Patient is now an adult with a stable course and stabilized psychiatric symptoms in the course of the disease. Works in a workshop facility, accommodated in residential facility.
6m→P→63→7→13→M		
ADHD, problems in school, behavioral problems		A: initially diagnosed and treated as ADHD by unconnected children psychiatry, massive behavior problems. Before inconspicuous infancy (except for a slight delay in speech development). Methylphenidate (to improve attention problems) without lasting success. Patient then reconsidered as “not trainable”. CE: discreet bradykinesia, discreet intention tremor. Fine motoric skill disturbance and increased muscle tone of the lower extremities. Afterward molecular genetic HD diagnosis. FD: pathological EEG without seizures. SM: Aggressive behavior towards other children and adults. Massive crimes documented by police with aggression at the age of six years (documented rape attempt on a girl, robbery on an elderly woman and theft of a handbag in the age of 12) led to in- patient diagnostics in a child and youth psychiatry. Persistent behavioral problems with short attention span, lack of controllability for own behavior and verbal aggression persisted. Diagnosis however led to correction of expectations and consequently to relaxing of the loaded situation. TA: risperidone (improved situation), zuclopenthixole (positive influence on irritability) stabilized conditions.
7f→P→85→6→9→P		
Motor abnormalities increasingly occurred on the left side	Epilepsy	A/CE: Expressive speech development delay (received speech therapy). Increasing dystonia of the left foot, three years after the first motoric symptoms and following genetic HD testing. Increasing lethargy in the course of disease, dystrophic (weight < 3 percentile, length < 3 percentile). Saccadic eye movement, chameleon tongue, dystonia of left foot, tremor, bradykinesia, increased muscle tone and reflexes. FD: pathological EEG. TA: venlafaxine or tetrabenazine suggested (lethargy). Various pharmaceutical trials (including L-dopa, amantadine, trihexyphenidyl) without success. CD: Tonic- clonic movement considered as a seizure and valproate used as an anticonvulsive. Dronabinol at request of mother improved dystonia.
8m→P→68→15→20→P		
Bradykinesia, progressive loss of concentration		A: positive family anamnesis, but initially no genetic testing wanted by patient and mother. TA: After several frustrating trials with pharmaceutical treatments, Deep brain stimulation (DBS): stereotactic implant of electrons in globus pallidus both sides. An initially setting effect with reduction of dystonia and rigor leading to a better body posture, better fine motor activity and less blepharospasm but worsening of gait and postural instability reported, in summary no significant objective long-term effect. Additional quetiapine, L-dopa/benserazide, pramipexole, seroquel. Fresurbin through PEG. Dopa- induced psychosis (optic and acoustic hallucination) lead to reduction of madopar. CD/SM: Hypersalivations and frequently bronchopneumonia. Organization of a school accompaniment.
9m→P→70→13→17→P		
Progressive movement disorders with hypokinetic- rigid aspects	Motoric axonal polyneuropathy DD: critical illness polyneuropathy	A: initially no genetic testing wanted by patient and mother (brother of case 8). TA/CD: Initially Madopar and memantine for cognitive symptoms afterwards DBS without beneficial effect, PEG with dislocation/ hematin vomiting, hypernatremia/ thrombopenia, respiratory decompensation. Urine, defecation-incontinence. Intermittent beneficial botulinum-toxin injection for treatment of dystonia in upper-extremity. Progressing exanthema (better after stopping of sifrol, Nexium, sirdalud). Amantadine, rotigotine lead to an improvement and better position of contract arms/ head. Due to ill skin loss of water with hypertonic dehydration. Topramax due to dystonia lead to an improvement of tremor. Additionally cannabinoids (Sativex-Spray©) leads to marked improvement of dystonia. SM: organization of a school accompaniment.
10f→J→61→20→23→P		
Psychiatric symptoms with attacks of suffocation and heart-pain without clinical correlate, depression	Sudden infantile death syndrome with reanimation	A: At the age of 23 already lived in a care home without HD diagnosis. Before multiple times inpatient in psychiatry because of panic attacks. Post-traumatic stress disorder (PTSD, no further circumstances described). Reported on unspecific described general pain of the whole body during clinical assessments. CE: stiff movement disorder, dysarthria. TA: Trials with baclofen, lorazepam, madopar, mirtazapine, seroquel, tolperisone, amantadine. L-dopa improved movement disorder with “more fluid”. mydocalm (pain) improved sleep, before suffering from nightmares. Stop of amantadine, instead memantine and artane (dystonic posture of the head) lead to a better movement of motoric and psychiatric situation.
11m→P→>60→8→11→M		
Motoric restlessness and multiple psychiatric problems		A/SM: Since early childhood psycho- social conflict situations and multiple continuous mistreatments due to father who made the patient alcohol- dependent at the age of two. Mistreated with cigarette pushing against the arm and aggressive behavior. Afterwards lived with grandfather (by care). TA: ritalin initially, later risperidone, valproate, tiapride CD: challenging loss of weight, swallowing and chewing difficulties.
12m→P→64→9→11→P		
Aggressive behavior and hyper movement of eyes	Epilepsy Amblyopia	A: Loss of concentration, difficulties finding words and loss of memory. A described reduction of intelligence. FD: MRI with bilateral signal increase Globus pallidus calcification and symmetric expansion of the lateral ventricle. SM: psycho-social conflict situation in family with massive problems: traumatic situations (saw brother drowning, diverse partners of mother mistreated the patient and mother). TA: valproate (Convulex) for epilepsy resulted also in less agitation and aggressive behavior after risperidone. CD: swallowing and chewing difficulties.
13m→P→Extended CAG, without reporting of exact repeat length→14→17→P		
Changing of speech and bradykinesia		A/CE: aggressive behavior and bradykinetic rigid movement disorder. FD: computed tomography (CT) with atrophy of Caput nuclei caudate and expansion of lateral ventricle front horns. SM: increased alcohol abuse since the age of 18, marihuana abuse (treated around the age of 15–17), worked in supervised workshop. TA: Rotigotine- pavement lead to improvement of movement. Bupropion (elontril) lead to an improvement of impulsive behavior, additionally Seroquel.
14m→P→Extended CAG, without reporting of exact repeat length→6→8→M		
Conflicts and aggressive behavior		CE: bradykinesia with worsening of movements. SM: Lived in a boarding school, before in a care family, aggressive behavior with impulsive outbursts. Nicotine abuse. TA: ebixia, lamictal, cipramil and requip (as dopaminergic stimulation) lead to an improvement of symptoms.
15f→J→52→19→23→M		
Dystonic-hypokinetic symptoms, loss of concentration	Bronchial asthma, atopic dermatitis	A/CE: Worsening of fine motor skills (writing) and loss of concentration. Drug abuse until the age of 23 (marihuana, PEP, cocaine), intermittent alcohol abuse and nicotine abuse. Stopped clinical evaluation and inpatient therapy herself because she already felt an improvement of symptoms. TA: Requip modutap lead to a better gang picture and better fine motoric skills. Did not take medication further. Second inpatient evaluation, again discharge against medical advice (she did not see an improvement). Berotec inhaler (if needed for distress), novaminsulfon (due to chronic pain), trial with tetrabenazine stopped due to a described “Heart pain”.
16m→J→Extended CAG, without reporting of exact repeat length→20→25→P		
Hyperkinesia motoric clumsiness	Drug-induced parkinsonism	A/CE: Molecular genetic testing due to hyperkinesia. Description of a rapidly massive worsening of chorea during 3-4 weeks. TA: Induced increasing of tiapride, swallowing problems, fatigue and no improvement of chorea, more a subjective worsening and parkinsonism. Because of the side effect and expected same side effects of other typical postsynaptic neuroleptics: Therapeutic target with tetrabenazine which improved symptoms very well (tiapride, tetrabenazine and trihexyphenidyl in combination) with three further stable years. CD/TA: at the age of 28 more dystonia and myocloniform hyperkinesia lead to a trial with valproate and reduced tiapride, tetrabenazine with satisfying improving of symptoms.
17f→J→Extended CAG, without reporting of exact repeat length→20→23→unknown, no HD family history		
Agitation, cognitive deficits	Emotionally unstable personality disorder	A/CE: Speech problems, swallowing problems, movement disorder in terms of gear insecurity, problems with coordinative movements. SM: Reporting on conflicts concerning the social background, lack of care. Aggressive and oppositional behavior, depression with suicidal ideation. Went to a special school, but without school-leaving certificate and lived in a residential facility because of integration problems. TA: Initial treatment with sulpiride, discontinuing of medication lead to an improvement. Lamictal induced because of fluctuation of affliction, amantadine lead to visual hallucination, theophylline, Broncho spray, Belladonysat additional.
18m→J→55→20→23→M		
Tremor		A/CE: Initially tremor, described by parents and patient. Intermittent suicidal thoughts, aggressive and irritable behavior. Posture and action tremor in clinical assessment, also tremor of the tongue. Only very slight intermittent hyperkinesia, more bradykinesia and rigid tone. TA: citalopram in combination with quetiapine and dopamine-agonist pramipexole initially in small doses due to danger of psychotic disorder lead to an improvement.
19f→J→53→20→22→P		
Motoric clumsiness	Epilepsy Hypothyroidism	A: massive psychiatric symptoms with depression, irritability, aggressive behavior, perseverative/obsessive behavior. FD: EEG with evidence of spike-wave-complexes left parietal. TA: Difficult pharmacological situation due to recurring unrest and fatigue: Trials with L-thyrox, levetiracetam, mirtazapine, nitoman, lamotrigine, zuclopenthixole (because of affective disorder and irritability). Challenging BOL hyperkinesia (improved after tetrabenazine), reduction of levetiracetam improved fatigue. Pharmaceutical trials with tiapride, sulpiride, gabapentin, pipamperone. CD/TA: Dysarthria, suicidal attempt, jumped from fifth floor with incomplete paraparesis. More myoclonic movement disorder in upper extremities lead to a treatment with valproate.
20f→P→n.d.→n. d.→16→P		
n.d.	Epilepsy Dystonic tetra paresis Anemia	CE: dystonia, hypokinetic movement disorder with spasticity. TA: Madopar lead initially to worsening, medication was stopped. Lioseral against spasticity lead to an improvement of symptoms. CD/TA: Tremor, increasing of creatine kinase and fever lead to the diagnosing of a malignant neuroleptic syndrome (MNS) which was stable after discontinuing the medication. Increase of spasticity and psychotic behavior lead to trials with tavor, cipramil, lioresal, seroquel, zuclopenthixole. Before massive psycho-motoric unrest with attacks of screaming. Intermittent infections (bronchopulmonary due to aspiration?). Trials with levodopa, oxazepam, diazepam, keppra, nexium, baclofen, seroquel, tolperisone, durogenesic patch, tizanidine. As needed: tavor (fear and restlessness), melperon (restlessness), diazepam (spasticity or seizure).
21f→J→60→20→22→P		
Psychiatric symptoms, depression. Hyperkinesia	Sterilization	CE: only slight hyperkinesia initially (trunk, extremities). FD: MRI with extension of the lateral ventricles and lateral ventricle anterior horns. CD: Massive loss of weight (20 kg in two years) and recurring infections. Changing of sleep- awake rhythm (trial with mirtazapine improved situation). Tiapride and tetrabenazine stabilized motoric and psychiatric situation.
22m→P→56→17→20→M		
Hyperkinesia and motoric clumsiness, depression	Thoracicosteochondrosis	CE: Hyperkinesia and a combination of dystonic components as part of the movement disorder. SM: finished school and professional training. FD: MRI with subcortical atrophy. CD/TA: Suicidal ideation (driving against a tree), self-harming behavior and sleep disorder. Mirtazapine, tiapride improved situation greatly. Increasing aggressive behavior lead to trials with citalopram, valproate, risperidone, arcoxia, memantine, zopiclone, pipamperone, elontril, tavor. Because of BOL- hyperkinesia botox was induced into the masseter. Disclaiming of food intake, massive perseverative behavior and decubitus during hospitalization. Changing of day/night rhythm = better after reduction of clozapine and increase of Haloperidol. Urine, defecation- incontinence.
23m→J→57→20→23→P		
Restlessness, worsening of fine motoric skills		CE/TA: Loss of concentration plus senso-motoric dysarthria. Hypokinetic- rigid symptoms with good response of levodopa, long lasting dopamine-agonist starting with pramipexole ret. CD/TA: Worsening of bradykinesia, tendency to fall. Trial with amantadine, quetiapine because of a sleep disorder, zopiclone as needed.
24f→J→66→19→21→unknown, no HD family history		
Hyperkinesia and restlessness.	Epilepsy with grand mal seizure	CE: Initially ocular interrupted pursuits, increased saccade latency and slow velocity. Fine motoric movement disorders with finger taps/ pronate supinate hands severe slowing and irregular. TA: Tetrabenazine even before diagnosis of HD, baclofen for dystonia. Diazepam for treatment of epilepsy. CD: suffered from slight intermittent chorea in combination with marked/ prolonged dystonia especially in upper extremities and markedly slow bradykinesia
25m→P→64→15→16→P		
Psychiatric symptoms	Allergic asthma	A/CE: Violent/aggressive behavior, apathy, perseverative, obsessive behavior. Chorea only slight/intermittent BOL/Trunk. Fine motoric skills difficulties, saccades and ocular pursuit. Previous suicidal attempts. TA: escitalopram (obsessive-compulsion) and quetiapine (mood stabilization) improved situation.
26m→P→67→9→11→n.d.		
n.d.	n.d.	n.d.
27f→J→58→20→n.d.→n.d.		
n.d.	n.d.	n.d.
28m→J→67→18→n.d.→n.d.		
n.d.	n.d.	n.d.
29m→J→53→18→n.d.→n.d.		
n.d.	n.d.	n.d.
30f→J→50→18→n.d.→n.d.		
n.d.	n.d.	n.d.
31f→P→77→12→n.d.→n.d.		
n.d.	n.d.	n.d.
32m→J→n.d.→18→n.d.→n.d.		
n.d.	n.d.	n.d.

Patients are itemised to: individual patient number (numeric) and female (f) or male (m) sex, pediatric (P) or juvenile (J) onset, CAG repeat- expansion length, age of onset (years), age of diagnosis (years), paternal (P) or maternal (M) inheritance. Description of predominant challenging situation classified according to anamnesis (A), clinical examination (CE), further diagnostics (FD), socio-medical aspects (SM), treatment attempt (TA) and course of disease (CD). Abbreviation n.d. (no given data).

**Table 2:** Demographics of the pediatric and juvenile HD cohort.

Demographics	Pediatric (*N* = 18)	Juvenile (*N* = 14)
Male/female sex (%)	72.2/27.8	42.9/57.1
CAG- repeat-expansion length	67.3 (SD 11.6, *n* = 14)	57.4, SD 5.6, *n* = 11)
Age of onset (AO) (years)	10.3 (SD 4.1, *n* = 16)	19.3 (SD 0.9, *n* = 14)
Age of diagnosis (AD) (years)	13.5 (SD 3.8, *n* = 17)	23.2 (SD 1.2, *n* = 9)
Mean time between AO and AD (years)	3.5 (SD 1.9, *n* = 15)	3.0 (SD 1.0, *n* = 9)
Paternal/maternal inheritance	68.8/ 31.2 (*n* = 16)	71.4/28.6 (*n* = 7)
